# A Bioacoustic Record of a Conservancy in the Mount Kenya Ecosystem

**DOI:** 10.3897/BDJ.4.e9906

**Published:** 2016-10-05

**Authors:** Ciira wa Maina, David Muchiri, Peter Njoroge

**Affiliations:** ‡ Department of Electrical and Electronic Engineering, Dedan Kimathi University of Technology, Nyeri, Kenya; § Dedan Kimathi University Wildlife Conservancy, Dedan Kimathi University of Technology, Nyeri, Kenya; | Ornithology Section, Department of Zoology, National Museums of Kenya, Nairobi, Kenya

**Keywords:** Conservation, Bioacoustics, Indicator taxa, Bird species, Raspberry Pi

## Abstract

**Background:**

Environmental degradation is a major threat facing ecosystems around the world. In order to determine ecosystems in need of conservation interventions, we must monitor the biodiversity of these ecosystems effectively. Bioacoustic approaches offer a means to monitor ecosystems of interest in a sustainable manner. In this work we show how a bioacoustic record from the Dedan Kimathi University wildlife conservancy, a conservancy in the Mount Kenya ecosystem, was obtained in a cost effective manner. A subset of the dataset was annotated with the identities of bird species present since they serve as useful indicator species. These data reveal the spatial distribution of species within the conservancy and also point to the effects of major highways on bird populations. This dataset will provide data to train automatic species recognition systems for birds found within the Mount Kenya ecosystem. Such systems are necessary if bioacoustic approaches are to be employed at the large scales necessary to influence wildlife conservation measures.

**New information:**

We provide acoustic recordings from the Dedan Kimathi University wildlife conservancy, a conservancy in the Mount Kenya ecosystem, obtained using a low cost acoustic recorder. A total of 2701 minute long recordings are provided including both daytime and nighttime recordings. We present an annotation of a subset of the daytime recordings indicating the bird species present in the recordings. The dataset contains recordings of at least 36 bird species. In addition, the presence of a few nocturnal species within the conservancy is also confirmed.

## Introduction

The world's biodiversity faces a number of threats including human encroachment into wildlife habitats and climate change. With a large number of species under threat, it is important to invest in conservation to ensure that these species are saved. However, due to limited resources it is important to target conservation efforts where they are most needed. To do this, it is important to collect relevant data from various ecosystems so as to determine those that are under threat and also those that have rich biodiversity. Efforts in this direction have led to the identification of biodiversity hotspots aimed at targeting conservation priorities (Myers et al. 2000). While the identification of these hotspots is an important step, conservation cannot be limited to just these regions (Kareiva and Marvier 2003). There is therefore a need to develop effective strategies to monitor a wide range of ecosystems so that conservation efforts can be effectively directed.

Current approaches to biodiversity assessment involve experts conducting surveys in the ecosystems of interest. While this approach is likely to lead to accurate measurement of species richness, it is expensive and cannot scale. Techniques such as rapid biodiversity assessment can be more widely applied because they limit the surveys to indicator taxa (Kerr et al. 2008) but they still require experts to conduct the surveys in the field. To obtain data on species richness at the scale needed to inform conservation efforts, it is necessary to automate the processes of data collection and annotation and develop methods to estimate species richness from these data. One step in this direction is the use of bioacoustic approaches to biodiversity monitoring where the sounds emitted by a wide range of living organisms are used to estimate species richness of the region from which the recordings were obtained (Sueur et al. 2008). Bioacoustic approaches to biodiversity monitoring have received considerable attention. They have been applied to monitor tropical ecosystems (Sueur et al. 2008) and to monitor species of interest including birds (Zwart et al. 2014, Ulloa et al. 2016), bats (Walters et al. 2012) and whales (Sciacca et al. 2015).

Bioacoustic approaches have several advantages over traditional surveys including 1) Acoustic recordings can be archived to serve as a permanent record of the ecosystem at a particular time. 2) Recording equipment can be used for long term monitoring. 3) It is straight forward to survey vocal nocturnal species using acoustic recorders. 4) Expert knowledge on the species of interest is not needed to mount the recorders. Despite these advantages, there are still a number of disadvantages including 1) Acoustic recorders generate a lot of data which are difficult to annotate and can be expensive to store. 2) Species that rarely vocalize will be disregarded in bioacoustic surveys. 3) Recording equipment can be expensive.

In order to increase the use of bioacoustic approaches in tropical ecosystems, it is necessary to address these shortcomings. A number of authors have demonstrated the use of low cost acoustic recorders for biodiversity monitoring (Farina et al. 2014). In addition, there are efforts to develop automatic species recognition systems for bird species which serve as useful indicator species in ecosystems of interest (Briggs et al. 2012, Stowell and Plumbley 2014​). It is important for research efforts to demonstrate the utility of acoustic recordings obtained in tropical ecosystems using low cost recorders for biodiversity assessment and to develop methods for automated species recognition based on acoustic recordings.

In this work we present a dataset of acoustic recordings obtained from the Dedan Kimathi University wildlife conservancy in central Kenya. This work is part of the Kenya Bioacoustics Project (https://sites.google.com/site/kenyabioacoustics/) which aims to use bioacoustic approaches for biodiversity monitoring within Kenya. The acoustic recordings in this dataset were obtained using a cheap recorder developed at the university. The recorder is based on the Raspberry Pi, a cheap microprocessor, connected to a cheap USB microphone. A number of recordings in the dataset are annotated by an expert ornithologist (PN, the third author) to indicate the bird species present in each recording and these provide a snap shot of the ecosystem during the duration of the study. This annotated dataset can be used to train automatic species recognition systems for use in other acoustic studies. Such a system has already been developed for the Hartlaub's Turaco (wa Maina 2016).

## Project description

### Study area description

The study was conducted at the Dedan Kimathi University Wildlife Conservancy (DeKUWC) located at 0°23'17.0"S, 36°57'43.2"E at an elevation of approximately 1800m (see Fig. 1). The conservancy covers an area of 120 Acres with three ecological zones namely open grassland, undisturbed indigenous forest and aquatic zones due to a permanent river that runs along its Northern boundary. The DeKUWC is located in the central part of Kenya and receives about 1000mm of rainfall annually. This is in two rainy seasons from mid March to May and October to November. There are two dry seasons from December to March and June to September. The conservancy is part of the Mount Kenya ecosystem with the Kabiruini forest bordering it to the North. The Kabiruini forest has suffered from human encroachment with quarrying and cattle grazing occurring within the forest. To the South, the conservancy is bordered by human settlements and a major highway (B5). See the map in Fig. 1 and the photographs in Fig. 2.

## Sampling methods

### Sampling description

Data collected in this study included point count data and acoustic recordings collected using a cheap microphone connected to a Raspberry Pi microprocessor. We (CwM and DM) performed point counts at twenty locations within the Dedan Kimathi University of Technology wildlife conservancy (DeKUWC) on two different days: 5th January, 2016 (10am to 12noon) and 28th January, 2016 (8am to 10am) (ten on each day). The points were separated by approximately 40 meters and birds seen or heard and judged to be within 20m of each location were recorded for ten minutes. Bird species identification was aided by the use of a guide book (Stevenson and Fanshawe 2002).

To collect the audio recordings, we used four acoustic recorders and these were left at locations near some of the point count locations. The recorders were left at ground level. A total of eight locations were sampled, four on each day. The 20 point count locations are labeled A-T and the acoustic recorder locations are labeled 1-8 as shown in Fig. 3. The recorders were left at these points for approximately 28 hours and were programmed to record for one minute at five minute intervals. This produced approximately 340 minute long recordings per site. We set the sampling rate of the recorders to 16kHz at 16 bit resolution. The recordings were saved in the uncompressed WAV format.

## Geographic coverage

### Description

The study was conducted at the Dedan Kimathi University Wildlife Conservancy (DeKUWC) located at 0°23'17.0"S, 36°57'43.2"E at an elevation of approximately 1800m.

## Taxonomic coverage

### Description

A total of 54 bird species were recorded during the study. Of these, 33 were recorded during the point counts and 36 identified using recordings of their vocalizations. 15 species were identified during both the point counts and using the audio recordings. The list of birds species identified during the study is shown in Table 1. This table includes a four-letter code used during annotation of the recordings. These codes were generated using the same rules used to generate the four-letter codes for North American birds (Klimkiewicz and Robbins 1978, Pyle and DeSante 2003). Photographs of a few of the birds are shown in Fig. 4.

**Point Count Data**

Table 2 shows the result of the point counts conducted on 5th January, 2016 while Table 3 shows the result of the point counts on 28th January, 2016. The number of individuals recorded at each location is shown.

**Audio Recordings**

Audio recordings were obtained using the Raspberry Pi based acoustic recorders from eight locations within the DeKUWC. A total of 2701 recordings were obtained. The locations are labeled 1-8 and are shown in the map on Fig. 3b. The acoustic recorder is described in the additional information section.

To determine the bird species present at the recording locations, a subset of the recordings in each location were carefully listened to and annotated. At each location around 20 recordings were annotated with the following information about each recording noted.

1. Date
2. Time
3. Location
4. Latitude
5. Longitude
6. Elevation in meters
7. Foreground species
8. Background species
9. Remarks

The foreground species included those species judged to be close to the microphone and clearly recorded. Background species on the other hand were species which could be identified but were judged to be far from the microphone. Remarks about the recording included information about any background noises and other features deemed noteworthy. For example, cow bells from the neighboring Kabiruini forest were heard on a number of recordings indicating presence of herders and their livestock in the forest. Engine noise was also prominent on recordings obtained near the road. It is important to monitor such sounds as they can be indicators of potential human-wildlife conflict and threats to biodiversity.

Table 4 shows a sample of the annotations for the audio recordings. The species are indicated using a four-letter code described in Pyle and DeSante (2003). The complete file is included in the supplementary material (Suppl. material 1). Each recording has a filename which indicates the location and time of the recording. For example the first file in Table 4, 1-2016-01-05-10-40-01, was recorded at location 1 on 5th January 2016 at 10:40 am.

**Recorded Species**

We obtained recordings from 36 of the 54 species observed in the study. Table 5 shows the number of foreground recordings per species while Table 6 shows the number of background recordings per species. Both lists are in descending order with the most recorded species appearing first. We see that the Yellow-whiskered Greenbul (Eurillas latirostris) is the most prominent species in the recordings. The Yellow-whiskered Greenbul is a very vocal species which makes it very easy to detect even during point counts. When making inferences about the abundance of bird species, it is important to take this into account to avoid over-estimating the abundance of vocal species.

**Spatial Distribution of Bird Species.**

Table 7 shows the number of foreground recordings per location for each of the species observed during the study. Table 8 shows the spatial distribution of all species identified in the recordings, both in the foreground and background of recordings. This allows us to infer the spatial distribution of species. We see that some species such as the Hartlaub's Turaco are highly concentrated in a single location while species such as the Yellow-whiskered Greenbul are more widespread.

## Usage rights

### Use license

Creative Commons Public Domain Waiver (CC-Zero)

## Data resources

### Data package title

DeKUWC Recordings

### Number of data sets

3

### Data set 1.

#### Data set name

DeKUWC Recorded Species

#### Data format

CSV

#### Number of columns

3

#### Download URL

https://doi.org/10.5061/dryad.69g60

#### Description

This file contains a list of the 54 bird species observed during the study.

**Data set 1.** 

Column label	Column description
Common Name	Species common name
Scientific Name	Species scientific name
Four Letter Code	Four letter code to identify the species

### Data set 2.

#### Data set name

Recordings Annotation

#### Data format

Excel

#### Number of columns

10

#### Download URL

https://doi.org/10.5061/dryad.69g60

#### Description

This file contains a list of the 2701 recordings obtained during the study from the eight recorder locations. Each location has a corresponding sheet in the Excel document. A subset of the 2701 recordings are annotated and for these recordings we have the following information:

**Data set 2.** 

Column label	Column description
Filename	File name of the mp3 file
Date	Date of recording
Time	Time of the recording
Location	Place the recording was taken
Latitude	Latitude of the location
Longitude	Longitude of the location
Elevation	Elevation above sea level of the location
Foreground Species	List of species in the foreground of the recording
Background Species	List of species in the foreground of the recording
Remarks	Any remarks on the recording

### Data set 3.

#### Data set name

Audio Recordings

#### Data format

MP3

#### Number of columns

1

#### Download URL

https://doi.org/10.5061/dryad.69g60

#### Description

The folder mp3/ contains MP3 files of the 2701 recordings obtained during the study.

**Data set 3.** 

Column label	Column description
Filename	File name of the MP3 file

## Additional information

### Discussion and Conclusion

We have presented a dataset of acoustic recordings obtained within the Dedan Kimathi Wildlife Conservancy in central Kenya. The DeKUWC is part of the Mount Kenya ecosystem which is an important ecological zone in Africa. In addition to being an important water tower, the Mount Kenya ecosystem is home to several important plant and animal species some of which are endemic to the region.

The recordings we present were obtained using a low cost recorder based on the Raspberry Pi microprocessor. The prototype cost approximately $100 and this allowed us to deploy four recorders at a time. The recordings obtained were of good enough quality to allow identification of bird species vocalizations and also other noise sources such as car engines and people. In addition, an initial attempt at automatic classification of a single species, the Hartlaub's Turaco, using these recordings has been successful (wa Maina 2016).

The study involved both point counts and acoustic recording and 54 bird species were observed. Of these, 33 were recorded during the point counts and 36 identified using recordings of their vocalizations. 15 species were identified during both the point counts and using the audio recordings. We see that the use of acoustic recordings allowed the identification of some species which were not observed during the point counts. This could be due to a number of reasons including 1) The acoustic recordings were obtained over the whole day. Thus if a bird was active outside the point count duration it was still captured by the acoustic recorder. 2) The bird vocalizations could be listened to several times to aid identification. On the other hand, a number of species were observed only during the point counts. These included raptors (Augur Buzzard) and species that are not very vocal such as the Black saw-wing. Vocal species such as the Speckled Mousebird recorded only in the point counts could be present in recordings that have not been annotated.

The acoustic recordings revealed the spatial distribution of bird species within the conservancy with some species such as the Yellow-whiskered Greenbul wide spread and others such as the Hartlaub's Turaco more concentrated in a few locations. With these data as a baseline, future studies can be used to monitor any changes to this spatial distribution and help to infer reasons for this change.

This dataset also reveals the effects of roads on wildlife populations. Engine noise is a prominent noise source in the recordings, particularly in the recordings obtained near the road. It was observed that the recorder nearest the major Nyeri-Nyahururu highway (B5) recorded the fewest number of species. As shown in Table 8 , only 7 species were recorded in both foreground and background of recordings at location 7 which is closest to the highway compared to 20 species recorded at the location with the highest number which was located further from the road, location 8. This confirms conclusions from other authors that roads have a major impact on wildlife populations (Votsi et al. 2012, Goodwin and Shriver 2010).

In conclusion, this study has provided a set of acoustic recordings collected using a cheap recorder which can be used to determine vocalizing species present in the recording and also to serve as useful data to train automatic species recognizers. It demonstrates that similar data collected over longer periods can be useful in aiding conservation efforts by effectively and cheaply monitoring ecosystems of interest.

### Acoustic Recorder

The recordings in this study were obtained using a Raspberry Pi (RPi) based recorder as shown in Fig. 5. The Raspberry Pi is a cheap credit card sized microprocessor that can be programmed like a desktop computer. In addition, the RPi can be connected to a number of sensors including microphones. The RPi's used in this study run the Raspbian operating system which is similar to Ubuntu https://www.raspberrypi.org. To obtain the recordings, we installed SoX which is an open source program for audio recording and processing http://sox.sourceforge.net/. It allows users to specify parameters such as the duration of the recording and sampling rate. In this study we used a sampling rate of 16kHz and the samples were stored using 16 bit resolution. The script used to set parameters for the SoX program *rec* is shown in the supplementary material (Suppl. material 2). After recording, the files were processed to ensure the maximum sample magnitude was unity. The recordings were stored using the WAV format. We also generated compressed MP3 files to reduce storage requirements.

The RPi recorders were powered using a 5V 6250 mAh battery bank similar to the one in Fig. 5a. This battery was able to power the recorder for approximately 28 hours with the recorder programmed to obtain a one minute recording every five minutes. The total cost of the prototype was approximately $100 which is significantly cheaper than most commercially available wildlife recorders such as the Song Meter from Wildlife Acoustics, Inc (http://www.wildlifeacoustics.com/). An itemized budget is included in the supplementary material (Suppl. material 3).

### Additional Species

In addition to bird species identified using their vocalizations. The dataset includes the vocalizations of other nocturnal creatures. These include crickets and tree hyraxes. Recordings 4-2016-01-06-00-40-02 and 4-2016-01-06-00-45-01 contain clear recordings of tree hyraxes.

## Supplementary Material

Supplementary material 1 Annotation File Data type: An Excel sheet with audio file description and annotation.
Brief description: This file contains the description of the 2701 audio files recorded at the DeKUWC. Of these, around 200 are annotated and this additional information is included.
File: oo_94637.xlsx Ciira wa Maina, Peter Njoroge

Supplementary material 2 Recording Script Data type: software
Brief description: This script is called to record the acoustic signals for one minute every five minutes. The SoX program rec performs the recording.
File: oo_94640.sh Ciira wa Maina

Supplementary material 3 Acoustic Recorder Cost Data type: Excel spreadsheet
Brief description: This file gives the cost of the components used to develop the acoustic recorder prototype.
File: oo_89872.xlsx Ciira wa Maina

## Figures and Tables

**Figure 1a.**
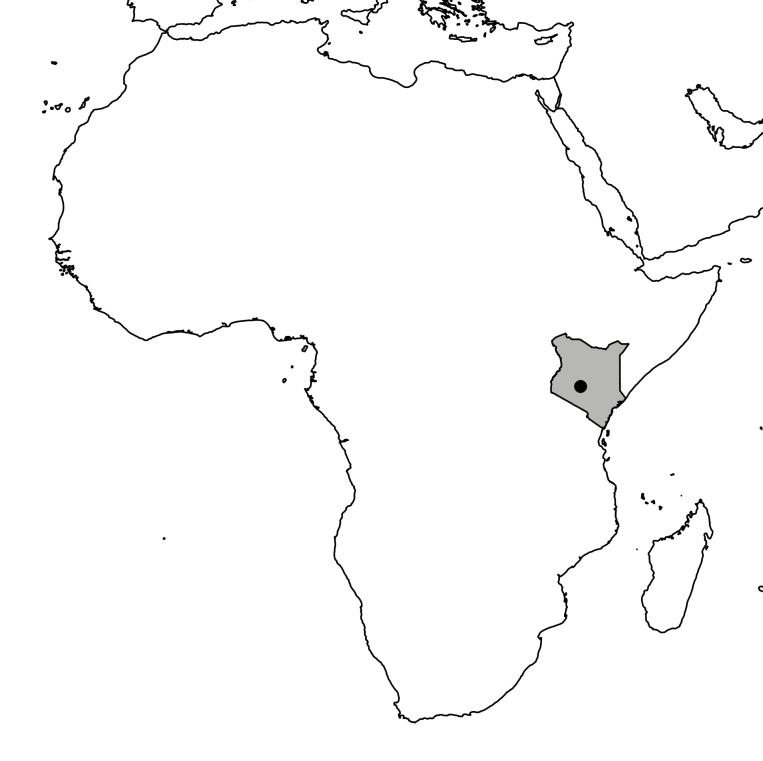


**Figure 1b.**
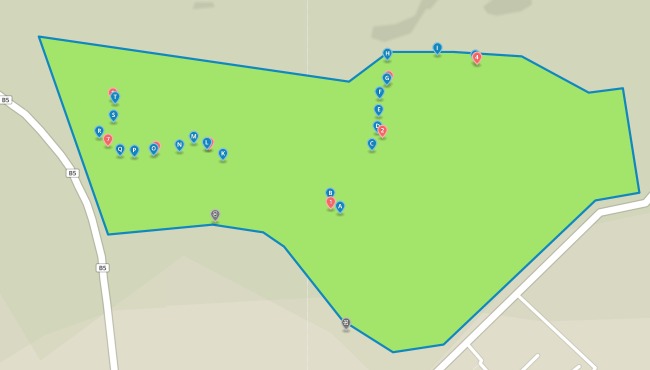


**Figure 2a.**
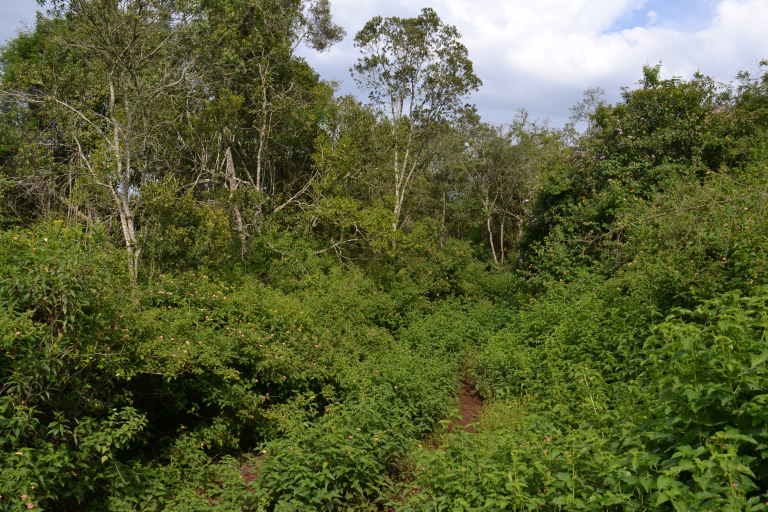


**Figure 2b.**
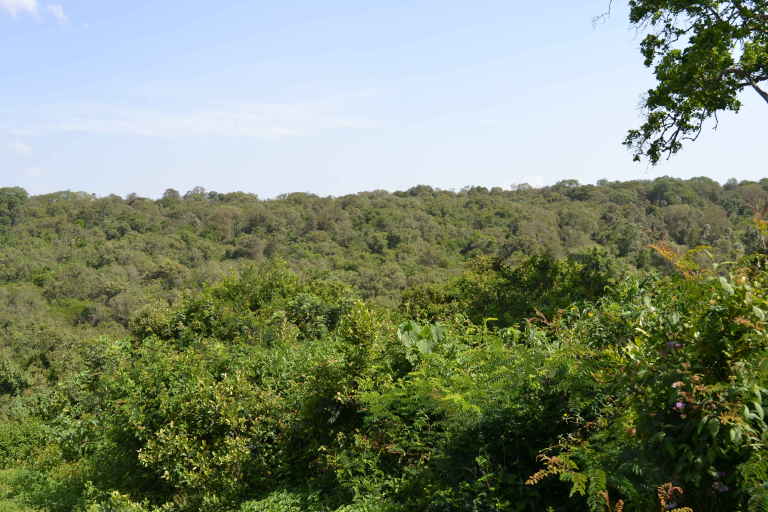


**Figure 3a.**
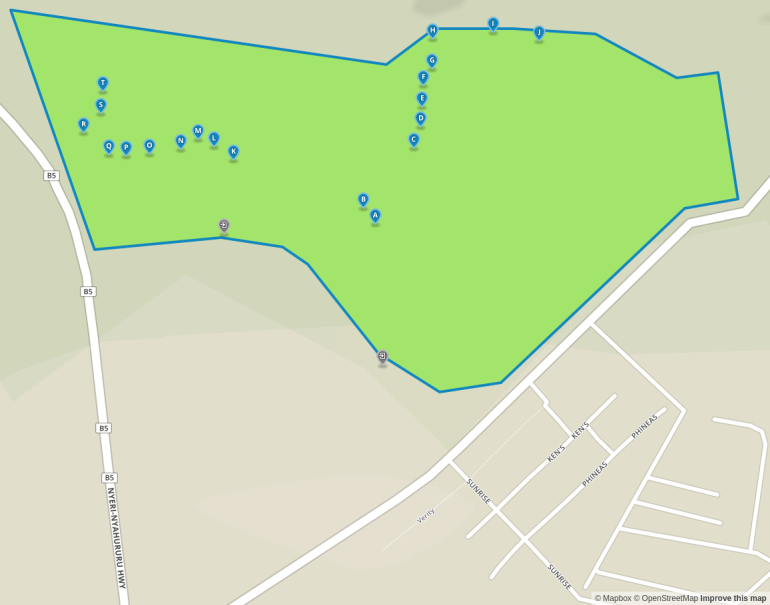


**Figure 3b.**
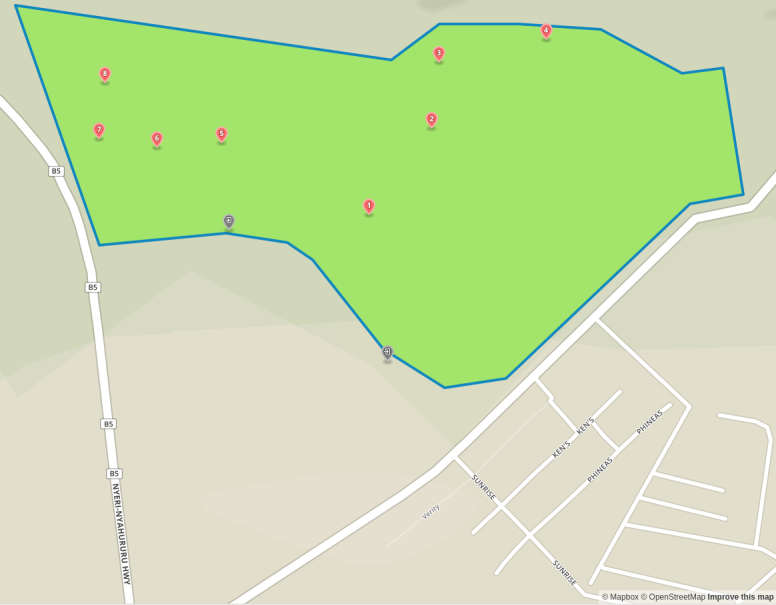


**Figure 4a.**
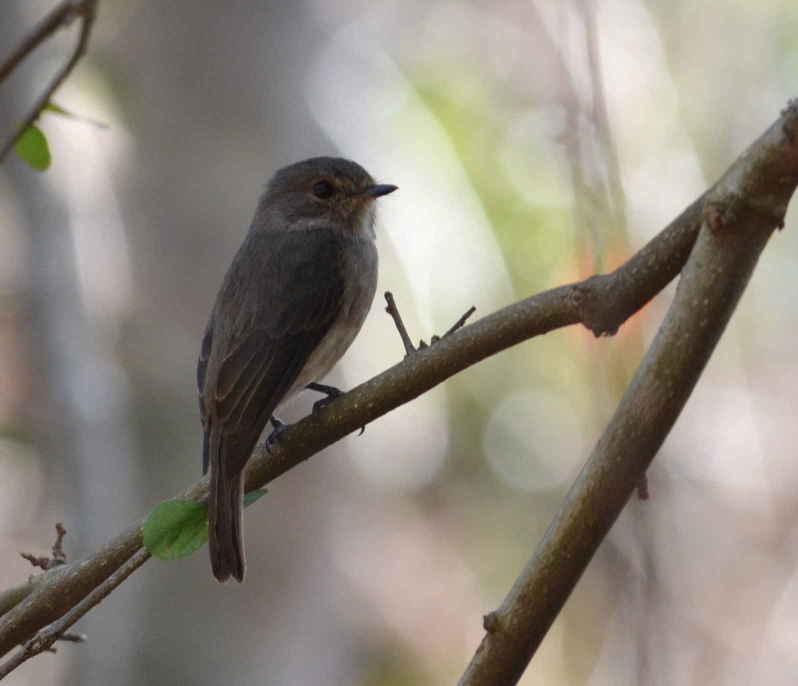
African Dusky Flycatcher

**Figure 4b.**
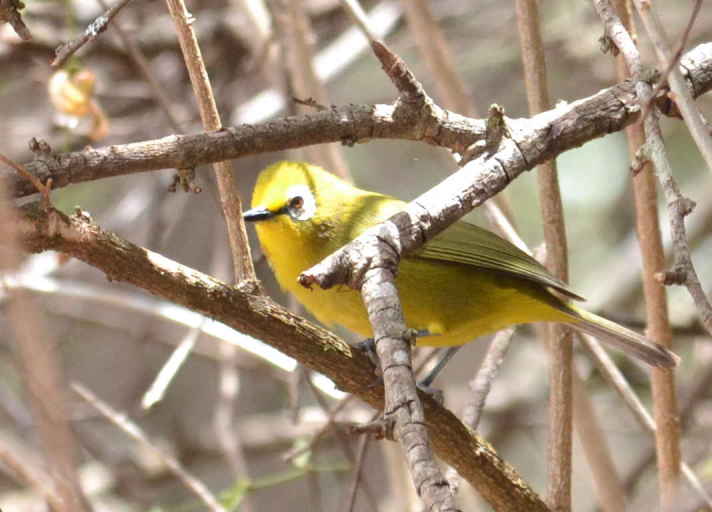
Montane White-eye

**Figure 4c.**
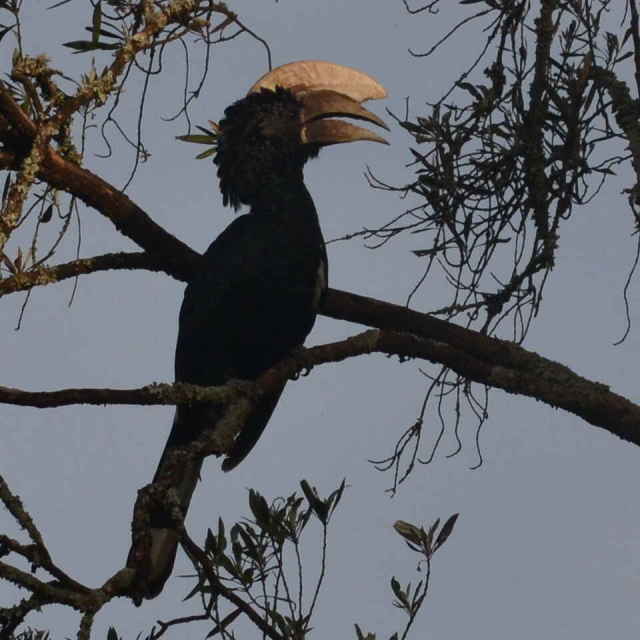
Silvery-cheeked Hornbill

**Figure 4d.**
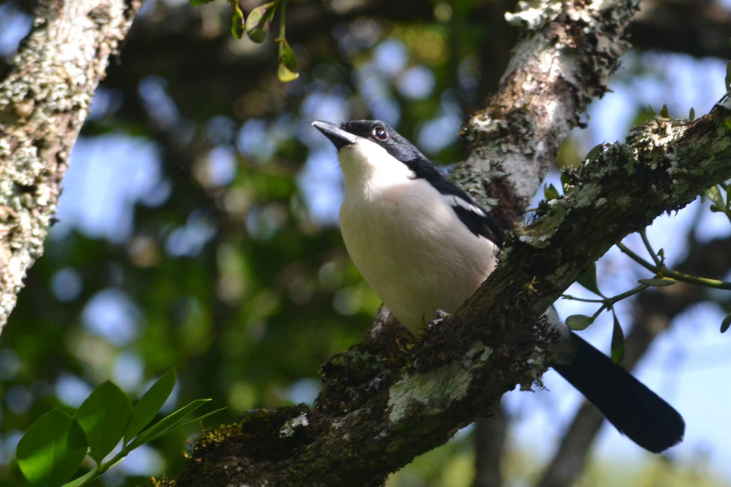
Tropical Boubou

**Figure 5a.**
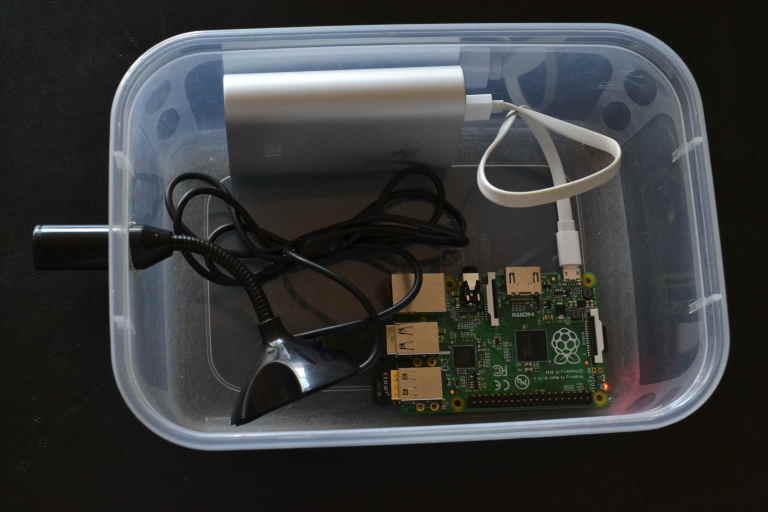


**Figure 5b.**
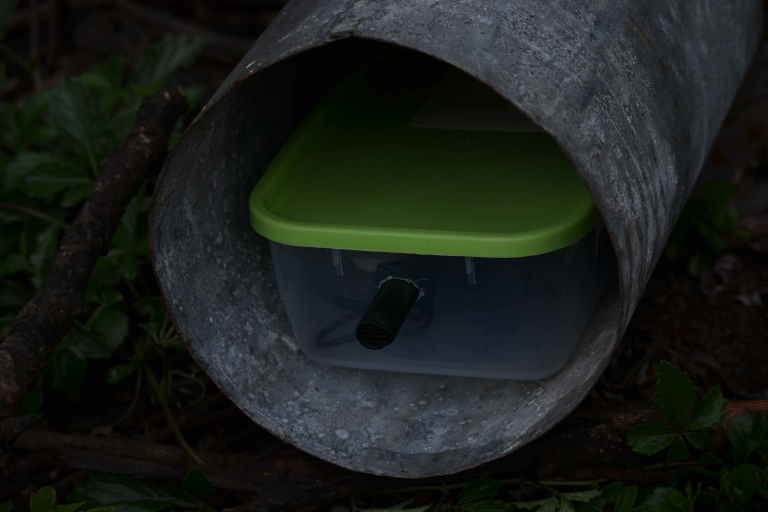


**Table 1.** Bird species identified during the study.

#	**Common Name**	**Scientific Name**	**Four-Letter Code**
1	Abyssinian Crimsonwing	*Cryptospiza salvadorii*	ABCR
2	African Dusky Flycatcher	*Muscicapa adusta*	ADFL
3	African Grey Flycatcher	*Melaenornis microrhynchus*	AGFL
4	African Paradise Flycatcher	*Terpsiphone viridis*	APFL
5	Augur Buzzard	*Buteo augur*	AUBU
6	Black-backed Puffback	*Dryoscopus cubla*	BBPU
7	Black-collared Apalis	*Oreolais pulcher*	BCAP
8	Black Cuckoo	*Cuculus clamosus*	BLCU
9	Black-headed Oriole	*Oriolus larvatus*	BHOR
10	Black Saw-wing	*Psalidoprocne pristoptera*	BLSW
11	Black-throated Wattle-eye	*Platysteira peltata*	BTWE
12	Blue-mantled Crested Flycatcher	*Trochocercus cyanomelas*	BMCF
13	Brown Woodland Warbler	*Phylloscopus umbrovirens*	BWWA
14	Cape Robin-Chat	*Cossypha caffra*	CARC
15	Chinspot Batis	*Batis molitor*	CHBA
16	Cinnamon-chested Bee-eater	*Merops oreobates*	CCBE
17	Collared Sunbird	*Hedydipna collaris*	COSU
18	Common Bulbul	*Pycnonotus barbatus*	COBU
19	Eastern Double-collared Sunbird	*Cinnyris mediocris*	EDCS
20	Emerald-spotted Wood-Dove	*Turtur chalcospilos*	ESWD
21	Eurasian Blackcap	*Sylvia atricapilla*	EUBL
22	Fork-tailed Drongo	*Dicrurus adsimilis*	FTDR
23	Golden-breasted Bunting	*Emberiza flaviventris*	GBBU
24	Grey Apalis	*Apalis cinerea*	GRAP
25	Grey-backed Camaroptera	*Camaroptera brevicaudata*	GBCA
26	Grey-capped Warbler	*Eminia lepida*	GCWA
27	Hadada Ibis	*Bostrychia hagedash*	HAIB
28	Hartlaub's Turaco	*Tauraco hartlaubi*	HATU
29	Holub's Golden Weaver	*Ploceus xanthops*	HGWE
30	Montane White-eye	*Zosterops poliogastrus*	MOWE
31	Mountain Yellow Warbler	*Iduna similis*	MYWA
32	Northern Double-collared Sunbird	*Cinnyris reichenowi*	NDCS
33	Olive Sunbird	*Cyanomitra olivacea*	OLSU
34	Olive Thrush	*Turdus olivaceus*	OLTH
35	Pied Crow	*Corvus albus*	PICR
36	Red-chested Cuckoo	*Cuculus solitarius*	RCCU
37	Red-headed Weaver	*Anaplectes rubriceps*	RHWE
38	Ring-necked Dove	*Streptopelia capicola*	RNDO
39	Ruppell's Robin-Chat	*Cossypha semirufa*	RURC
40	Silvery-cheeked Hornbill	*Bycanistes brevis*	SCHO
41	Southern Black Flycatcher	*Melaenornis pammelaina*	SBFL
42	Speckled Mousebird	*Colius striatus*	SPMO
43	Spectacled Weaver	*Ploceus ocularis*	SPWE
44	Spot-flanked Barbet	*Tricholaema lacrymosa*	SFBA
45	Tambourine Dove	*Turtur tympanistria*	TADO
46	Tawny-flanked Prinia	*Prinia subflava*	TFPR
47	Tropical Boubou	*Laniarius major*	TRBO
48	Variable Sunbird	*Cinnyris venustus*	VASU
49	White-browed Robin-Chat	*Cossypha heuglini*	WBRC
50	White-eyed Slaty Flycatcher	*Melaenornis fischeri*	WESF
51	Yellow Bishop	*Euplectes capensis*	YEBI
52	Yellow-breasted Apalis	*Apalis flavida*	YBAP
53	Yellow-rumped Tinkerbird	*Pogoniulus bilineatus*	YRTI
54	Yellow-whiskered Greenbul	*Eurillas latirostris*	YWGR

**Table 2.** Results of the point count on 5th January, 2016. The number of individuals (NOI) observed at various locations is indicated.

#	**Species**	**Point Count Location**										**NOI**
		**A**	**B**	**C**	**D**	**E**	**F**	**G**	**H**	**I**	**J**	
1	Red-headed Weaver	2										2
2	African Paradise Flycatcher	1	1									2
3	Cinnamon-chested Bee-eater	1										1
4	Common Bulbul		3	1	1		1		1	1	3	11
5	Black-collared Apalis		1									1
6	Black-backed Puffback		1									1
7	Yellow-whiskered Greenbul			1	1			1	1		1	5
8	Variable Sunbird			1	1	1					1	4
9	Tropical Boubou			1								1
10	African Golden-breasted Bunting			1								1
11	Grey-backed Camaroptera			1			1	1				3
12	Eurasian Blackcap				1							1
13	Yellow-breasted Apalis					1						1
14	Ring-necked Dove						1					1
15	Yellow-rumped Tinkerbird							2		1		3
16	Augur Buzzard								1			1
17	African Dusky Flycatcher								1			1
18	Grey Apalis									1		1
19	Spectacled Weaver									1		1
20	Tambourine Dove										1	1
21	Eastern Double-collared Sunbird										1	1
22	Chinspot Batis										1	1
23	Southern Black Flycatcher										1	1
24	Speckled Mousebird										4	4
25	Olive Thrush										1	1
26	African Grey Flycatcher										1	1
	**Number of Species**	3	4	6	4	2	3	3	4	4	10	

**Table 3.** Results of the point count on 28th January, 2016. The number of individuals (NOI) observed at various locations is indicated.

#	**Species**	**Point Count Location**										**NOI**
		**K**	**L**	**M**	**N**	**O**	**P**	**Q**	**R**	**S**	**T**	
1	Tropical Boubou	2	1				1	4				8
2	Grey-backed Camaroptera	2	1	1	1	1	3	2	2	1	1	15
3	Tawny-flanked Prinia	2										2
4	Yellow-whiskered Greenbul	1	2				1	2			1	7
5	Variable Sunbird	3	2		1			1		3	1	11
6	Common Bulbul	1		1	1		1		1	1		6
7	Holub's Golden Weaver		1									1
8	White-eyed Slaty Flycatcher			1								1
9	Ring-necked Dove			1								1
10	Chinspot Batis				1							1
11	Yellow-rumped Tinkerbird							1				1
12	Yellow-breasted Apalis								1			1
13	Silvery-cheeked Hornbill									6		6
14	Black-backed Puffback									1		1
15	Collared Sunbird									1		1
16	Montane White-eye									2	1	3
17	Black Saw-wing										1	1
	**Number of Species**	6	5	4	4	1	4	5	3	7	5	

**Table 4.** Annotation of audio recordings obtained at the DeKUWC (FS= Foreground Species, BS= Background Species)

**Filename**	**FS**	**BS**	**Remarks**
1-2016-01-05-10-40-01	YWGR	GBCA	Engine noise in the background
1-2016-01-05-11-10-01	TRBO	HATU	
1-2016-01-05-12-35-01	GBCA		Sound of engine; crow and insect in the background
1-2016-01-05-12-40-01	GBCA	TADO;HATU	Engine noise in the background
1-2016-01-05-13-20-01	COBU	YRTI	
1-2016-01-05-13-40-01	GBCA	TADO	
1-2016-01-06-06-30-01	GBCA	YWGR	Robin-Chat singing in the background
1-2016-01-06-06-35-01	YWGR	OLTH;HATU	
1-2016-01-06-06-40-02	GBCA	COBU;HAIB	
1-2016-01-06-07-00-01	GBCA;BBPU;PICR		
1-2016-01-06-07-35-01	BBPU;ABCR;BHOR		
1-2016-01-06-07-40-01	GBCA	YRTI	
1-2016-01-06-08-05-01	COBU;YWGR;SCHO		
1-2016-01-06-08-55-02	GBCA	BBPU;TRBO	
1-2016-01-06-09-30-01	HAIB		
1-2016-01-06-09-40-02	GBCA	BWWA;YRTI	
1-2016-01-06-10-45-01	YRTI	TRBO;GBCA;BWWA	
1-2016-01-06-12-30-01	FTDR	YRTI	
1-2016-01-06-14-05-01	GBCA;YRTI		
1-2016-01-06-14-10-01	YWGR;FTDR		

**Table 5.** Number of foreground recordings per species.

**Position**	**Common Name**	**Scientific Name**	**Number of Recordings**
1	Yellow-whiskered Greenbul	*Eurillas latirostris*	88
2	Grey-backed Camaroptera	*Camaroptera brevicaudata*	52
3	Hartlaub's Turaco	*Tauraco hartlaubi*	30
4	Yellow-rumped Tinkerbird	*Pogoniulus bilineatus*	29
5	Tambourine Dove	*Turtur tympanistria*	19
6	Tropical Boubou	*Laniarius major*	17
7	Black-backed Puffback	*Dryoscopus cubla*	11
8	Silvery-cheeked Hornbill	*Bycanistes brevis*	7
9	Common Bulbul	*Pycnonotus barbatus*	5
10	Ruppell's Robin-Chat	*Cossypha semirufa*	4
11	Olive Thrush	*Turdus olivaceus*	3
12	Fork-tailed Drongo	*Dicrurus adsimilis*	3
13	Collared Sunbird	*Hedydipna collaris*	3
14	Black-headed Oriole	*Oriolus larvatus*	2
15	African Paradise Flycatcher	*Terpsiphone viridis*	2
16	Brown Woodland Warbler	*Phylloscopus umbrovirens*	2
17	Red-chested Cuckoo	*Cuculus solitarius*	2
18	Olive Sunbird	*Cyanomitra olivacea*	2
19	Chinspot Batis	*Batis molitor*	1
20	Blue-mantled Crested Flycatcher	*Trochocercus cyanomelas*	1
21	African Dusky Flycatcher	*Muscicapa adusta*	1
22	Black-throated Wattle-eye	*Platysteira peltata*	1
23	Cape Robin-Chat	*Cossypha caffra*	1
24	Hadada Ibis	*Bostrychia hagedash*	1
25	Abyssinian Crimsonwing	*Cryptospiza salvadorii*	1
26	Pied Crow	*Corvus albus*	1
27	White-browed Robin-Chat	*Cossypha heuglini*	1
28	Variable Sunbird	*Cinnyris venustus*	1

**Table 6.** Number of background recordings per species.

**Position**	**Common Name**	**Scientific Name**	**Number of Recordings**
1	Yellow-whiskered Greenbul	*Eurillas latirostris*	59
2	Hartlaub's Turaco	*Tauraco hartlaubi*	38
3	Tropical Boubou	*Laniarius major*	32
4	Grey-backed Camaroptera	*Camaroptera brevicaudata*	32
5	Yellow-rumped Tinkerbird	*Pogoniulus bilineatus*	31
6	Tambourine Dove	*Turtur tympanistria*	29
7	Black-backed Puffback	*Dryoscopus cubla*	10
8	Brown Woodland Warbler	*Phylloscopus umbrovirens*	6
9	Silvery-cheeked Hornbill	*Bycanistes brevis*	6
10	Black-headed Oriole	*Oriolus larvatus*	5
11	Collared Sunbird	*Hedydipna collaris*	5
12	Common Bulbul	*Pycnonotus barbatus*	4
13	Olive Thrush	*Turdus olivaceus*	4
14	Chinspot Batis	*Batis molitor*	3
15	African Paradise Flycatcher	*Terpsiphone viridis*	2
16	Northern Double-collared Sunbird	*Cinnyris reichenowi*	1
17	Emerald-spotted Wood-Dove	*Turtur chalcospilos*	1
18	Cape Robin-Chat	*Cossypha caffra*	1
19	Grey-capped Warbler	*Eminia lepida*	1
20	Black-throated Wattle-eye	*Platysteira peltata*	1
21	Black Cuckoo	*Cuculus clamosus*	1
22	Mountain Yellow Warbler	*Iduna similis*	1
23	Hadada Ibis	*Bostrychia hagedash*	1
24	Olive Sunbird	*Cyanomitra olivacea*	1
25	Yellow-breasted Apalis	*Apalis flavida*	1
26	Yellow Bishop	*Euplectes capensis*	1
27	Spot-flanked Barbet	*Tricholaema lacrymosa*	1
28	Variable Sunbird	*Cinnyris venustus*	1

**Table 7.** Spatial distribution of foreground species. The number of foreground recordings per location for each of the species is indicated.

#	**Species**		**Recorder Location**							
	**Common Name**	**Scientific Name**	**1**	**2**	**3**	**4**	**5**	**6**	**7**	**8**
1	Abyssinian Crimsonwing	*Cryptospiza salvadorii*	1	0	0	0	0	0	0	0
2	African Dusky Flycatcher	*Muscicapa adusta*	0	0	0	0	0	1	0	0
3	African Paradise Flycatcher	*Terpsiphone viridis*	0	1	0	1	0	0	0	0
4	Black-backed Puffback	*Dryoscopus cubla*	2	3	0	2	0	4	0	0
5	Black-headed Oriole	*Oriolus larvatus*	1	0	0	0	0	0	0	1
6	Black-throated Wattle-eye	*Platysteira peltata*	0	0	0	1	0	0	0	0
7	Blue-mantled Crested Flycatcher	*Trochocercus cyanomelas*	0	0	0	0	0	0	0	1
8	Brown Woodland Warbler	*Phylloscopus umbrovirens*	0	1	0	0	0	0	0	1
9	Cape Robin-Chat	*Cossypha caffra*	0	0	0	0	0	1	0	0
10	Chinspot Batis	*Batis molitor*	0	0	0	0	0	0	0	1
11	Collared Sunbird	*Hedydipna collaris*	0	0	1	1	0	0	0	1
12	Common Bulbul	*Pycnonotus barbatus*	2	1	1	1	0	0	0	0
13	Fork-tailed Drongo	*Dicrurus adsimilis*	2	1	0	0	0	0	0	0
14	Grey-backed Camaroptera	*Camaroptera brevicaudata*	10	12	1	1	7	13	1	7
15	Hadada Ibis	*Bostrychia hagedash*	1	0	0	0	0	0	0	0
16	Hartlaub's Turaco	*Tauraco hartlaubi*	0	1	7	20	0	1	0	1
17	Olive Sunbird	*Cyanomitra olivacea*	0	0	0	1	0	0	0	1
18	Olive Thrush	*Turdus olivaceus*	0	0	0	0	0	1	0	2
19	Pied Crow	*Corvus albus*	1	0	0	0	0	0	0	0
20	Red-chested Cuckoo	*Cuculus solitarius*	0	0	0	0	1	1	0	0
21	Ruppell's Robin-Chat	*Cossypha semirufa*	0	1	0	0	0	1	1	1
22	Silvery-cheeked Hornbill	*Bycanistes brevis*	1	0	0	0	1	1	0	4
23	Tambourine Dove	*Turtur tympanistria*	0	1	9	9	0	0	0	0
24	Tropical Boubou	*Laniarius major*	1	3	2	2	1	8	0	0
25	Variable Sunbird	*Cinnyris venustus*	0	1	0	0	0	0	0	0
26	White-browed Robin-Chat	*Cossypha heuglini*	0	0	0	0	0	0	0	1
27	Yellow-rumped Tinkerbird	*Pogoniulus bilineatus*	2	3	5	10	3	6	0	0
28	Yellow-whiskered Greenbul	*Eurillas latirostris*	4	18	17	18	17	0	0	14
	**Number of Species**		12	13	8	12	6	11	2	13

**Table 8.** Spatial distribution of species in both foreground and background recordings. The number of recordings per location for each of the species is indicated.

#	**Species**		**Recorder Location**							
	**Common Name**	**Scientific Name**	**1**	**2**	**3**	**4**	**5**	**6**	**7**	**8**
1	Abyssinian Crimsonwing	*Cryptospiza salvadorii*	1	0	0	0	0	0	0	0
2	African Dusky Flycatcher	*Muscicapa adusta*	0	0	0	0	0	1	0	0
3	African Paradise Flycatcher	*Terpsiphone viridis*	0	1	0	3	0	0	0	0
4	Black-backed Puffback	*Dryoscopus cubla*	5	4	0	2	1	9	0	0
5	Black Cuckoo	*Cuculus clamosus*	0	0	1	0	0	0	0	0
6	Black-headed Oriole	*Oriolus larvatus*	1	0	0	0	2	2	1	1
7	Black-throated Wattle-eye	*Platysteira peltata*	0	0	0	1	1	0	0	0
8	Blue-mantled Crested Flycatcher	*Trochocercus cyanomelas*	0	0	0	0	0	0	0	1
9	Brown Woodland Warbler	*Phylloscopus umbrovirens*	2	1	1	0	3	0	0	1
10	Cape Robin-Chat	*Cossypha caffra*	0	0	0	0	0	1	0	1
11	Chinspot Batis	*Batis molitor*	0	1	0	1	1	0	0	1
12	Collared Sunbird	*Hedydipna collaris*	0	0	1	1	1	1	2	2
13	Common Bulbul	*Pycnonotus barbatus*	4	1	1	1	0	1	0	1
14	Emerald-spotted Wood-Dove	*Turtur chalcospilos*	0	0	0	0	0	1	0	0
15	Fork-tailed Drongo	*Dicrurus adsimilis*	2	1	0	0	0	0	0	0
16	Grey-backed Camaroptera	*Camaroptera brevicaudata*	15	15	8	7	11	14	4	10
17	Grey-capped Warbler	*Eminia lepida*	0	0	0	0	0	0	0	1
18	Hadada Ibis	*Bostrychia hagedash*	2	0	0	0	0	0	0	0
19	Hartlaub's Turaco	*Tauraco hartlaubi*	3	7	17	23	9	1	0	8
20	Mountain Yellow Warbler	*Iduna similis*	0	0	0	0	0	1	0	0
21	Northern Double-collared Sunbird	*Cinnyris reichenowi*	0	0	0	0	0	0	0	1
22	Olive Sunbird	*Cyanomitra olivacea*	0	0	0	1	0	0	0	2
23	Olive Thrush	*Turdus olivaceus*	2	0	0	0	0	3	0	2
24	Pied Crow	*Corvus albus*	1	0	0	0	0	0	0	0
25	Red-chested Cuckoo	*Cuculus solitarius*	0	0	0	0	1	1	0	0
26	Ruppell's Robin-Chat	*Cossypha semirufa*	0	1	0	0	0	1	1	1
27	Silvery-cheeked Hornbill	*Bycanistes brevis*	1	0	0	0	2	2	2	6
28	Spot-flanked Barbet	*Tricholaema lacrymosa*	0	1	0	0	0	0	0	0
29	Tambourine Dove	*Turtur tympanistria*	4	12	16	15	0	1	0	0
30	Tropical Boubou	*Laniarius major*	3	6	4	12	4	18	1	1
31	Variable Sunbird	*Cinnyris venustus*	0	1	0	0	0	0	0	0
32	White-browed Robin-Chat	*Cossypha heuglini*	0	0	0	0	0	0	0	1
33	Yellow Bishop	*Euplectes capensis*	0	0	0	0	0	0	0	1
34	Yellow-breasted Apalis	*Apalis flavida*	0	1	0	0	0	0	0	0
35	Yellow-rumped Tinkerbird	*Pogoniulus bilineatus*	8	10	9	14	8	8	0	3
36	Yellow-whiskered Greenbul	*Eurillas latirostris*	5	22	21	30	24	17	7	21
	**Number of Species**		16	16	10	13	13	18	7	20
